# Effect of P_2_O_5_ and Na_2_O on the Solubility of Molybdenum and Structural Features in Borosilicate Glass

**DOI:** 10.3390/ma15155464

**Published:** 2022-08-08

**Authors:** Hao Liu, Yongchang Zhu, Jichuan Huo, Zhu Cui, Xingquan Zhang, Qin Jiang, Debo Yang, Baojian Meng

**Affiliations:** 1 State Key Laboratory of Environment-Friendly Energy Materials, School of Materials and Chemistry, Southwest University of Science and Technology, Mianyang 621010, China; 2 China Building Materials Academy, Beijing 100024, China; 3 Fundamental Science on Nuclear Wastes and Environmental Safety Laboratory, Southwest University of Science and Technology, Mianyang 621010, China

**Keywords:** HLW, molybdenum, yellow phase, borosilicate glass

## Abstract

In this paper, the effect of doping phosphorus in a borosilicate glass matrix to improve the solubility of Mo was investigated by X-ray diffraction (XRD), Raman, and solid-state nuclear magnetic resonance (NMR) spectroscopy, and the effectiveness of Na content on P species inhibiting the growth of the crystallization of Mo was assessed. The results indicate that phosphate-doped borosilicate glass can host 4 mol% of Mo, and that such a borosilicate glass matrix could only accommodate 1 mol% of Mo without phosphate doping. The effectiveness of phosphorus may be correlated with the Na content in borosilicate glass, and a high Na content borosilicate glass matrix requires more P doping to accommodate Mo. In addition, incorporating large amounts of P can compromise the aqueous durability of the glass matrix.

## 1. Introduction

Some countries, such as China, France, and the UK, have adopted the closed nuclear fuel cycle technology route, which features the reprocessing of spent nuclear fuel (SNF) to recover transuranium elements (TRUs) such as uranium (U) and plutonium (Pu) [1,2,3,4,5]. The current most mature plutonium uranium redox extraction (Purex) process is used in commercial nuclear fuel recycling, and a highly level waste (HLW) from this step is rich in many types of fission products, corrosion products, remaining TRUs, and the solvent that is added during reprocessing [4,5,6,7,8]. Furthermore, vitrification is the only industrial technology to immobilize HLW [9]. Borosilicate glass is a favorable matrix because of its good durability, irradiation resistance, and high capacity of most nonvolatile elements in HLW [1,2,9,10]. However, it also faces complex problems in practical applications.

Borosilicate glass has a low solubility for some key waste components in HLW [1,11]. For instance, the molybdate (Mo) loading limit in conventional borosilicate glasses is less than 1.5 mol% [2,12,13]. This can be explained by the high field strength of Mo^6+^ (1.89–1.935 Å^−2^) [14]. Many studies indicate that Mo^6+^ induces short-range ordering of surrounding oxygens, which leads to Mo predominantly occurring as isolated [MoO_4_]^2−^ tetrahedra in alkali and alkaline earth-rich depolymerized regions of the borosilicate glass structure and are not linked directly to the glass network [1,2,15,16,17]. Thus, excess Mo tends to experience phase separation in the form of crystalline molybdates (yellow phase) from waste-immobilized glass [2,18,19]. Furthermore, the yellow phase may contain radioactive elements (^137^Cs or ^90^Sr) and dissolve easily in water [17,20]. Mo is one of the main fission products from HLW stored in China, France, etc. [9,21,22,23]. Therefore, the extent to which Mo solubility (S_Mo_) actually limits the total loading of waste in the borosilicate glass. In terms of improving S_Mo_, various cations, such as lanthanides, are added to the borosilicate glass. As lanthanides are present in the same area with [MoO_4_]^2−^, their presence may affect local charge compensation in the region surrounding [MoO_4_]^2−^, thus inhibiting Mo crystallization. Previous investigations have proved the beneficial effect of Nd_2_O_3_ on S_Mo_ in borosilicate glass [5,13,24,25,26]. However, the addition of phosphorus (P) is different from lanthanides.

Recent studies indicate that the addition of phosphorus also enhances S_Mo_ in silicate and borosilicate glass [5,18,27]. Unlike the effect of doping with normal cations, the successful integration of Mo in the right proportion of the phosphate network occurs through P–O–Mo bonds in silicate–phosphate glass, instead of only forming isolated [MoO_4_]^2−^; in other words, the formation of P–O–Mo bonds probably is also one of the keys to improving S_Mo_ in borosilicate glass by doping with P [1,18,27,28]. However, P can be used as both network formers and phosphate species in borosilicate glass, the ratio of which depends strongly on the glass composition. Literature data report that in sodium borosilicate glass with large Na_2_O, most of the P forms isolated P_2_O_7_^4−^ and PO_4_^3−^ units, and, conversely, a larger proportion of P would be bonded to the glassy network in the form of P–O–Al and P–O–B bonds [29,30,31,32,33,34,35]. Thus, the relative quantity of P–O–Mo may be related to the glass composition of the borosilicate glass as well.

To the best of our knowledge, the effect of Na_2_O content on S_Mo_ and structural features in phosphorus-doped borosilicate glass remains unclear. Moreover, excessive levels of P doping may compromise the matrix [36,37]. This knowledge may help researchers to assess the doping proportion and effectiveness of P in borosilicate glass for improving Mo vitrification. In this paper, the SiO_2_–B_2_O_3_–Na_2_O–P_2_O_5_ and SiO_2_–B_2_O_3_–Na_2_O glass series were used to investigate this phenomenon. The structural examinations of the glass were based on Raman spectra and solid-state nuclear magnetic resonance (NMR) spectroscopy. The aqueous durability of samples was assessed by product consistency tests (PCTs).

## 2. Experimental

### 2.1. Sample Preparation

Glass with the nominal compositions listed in Table 1 were synthesized by analytical reagents of SiO_2_ (Sinopharm Group; ≥99%), H_3_BO_3_ (Sinopharm Group; ≥99.5%), Na_2_CO_3_ (Sinopharm Group; ≥99.5%), MoO_3_ (Sinopharm Group; ≥99.9%), and NH_4_H_2_PO_4_ (Sinopharm Group; ≥99.5%) at the laboratory scale (100 g). The mixed batches were decarbonated at 600 °C for 2 h in corundum crucibles and melted at 1200 °C for 2 h. Quenching over a preheated graphite sheet afterward was conducted to obtain glass.

In the Mz series (z = [Na_2_O]), the Na content was changed by gradually replacing SiO_2_ through Na_2_O while keeping the B_2_O_3_ concentration constant (19.32 mol%), which is due to the strong influence of B_2_O_3_ on the crystallization of molybdate [15,38]. NBS-4P-4Mo was selected as the reference glass for the Mz series, with the same composition as M24.

### 2.2. Measurements

X-ray diffraction (XRD) patterns of the samples were measured using a Bruker D8 Endeavor X-ray diffractometer (Cu-Kα radiation), operating at 40 kV and 40 mA, ranging from 10° to 80°. Raman spectra were collected by a Renishaw InVia Raman spectrometer with a CCD detector in the range of 200–2000 cm^−1^. The powder samples of particle size less than 200 mesh were measured using the synchronous thermal analyzer (METTLER TOLEDO TGA/DSC3+) to record the DSC curves in the range of 50–1000 °C at the heated rate of 20 °C/min in air. ^31^P MAS NMR spectra were carried out on a JNM-ECZ600R spectrometer at a frequency of 242.95 MHz. The chemical spectra were referenced using 85% H_3_PO_4_, and the 90° applied pulse lengths were 2 μs. The compositions of glass samples were measured by X-ray fluorescent spectrum (XRF)

The PCT test was carried out in sealed PTFE reactors housed in an oven at 90 °C. The glass particles between 100 and 200 meshes were ultrasonically cleaned with anhydrous alcohol and then dried, and the specific surface area was tested with a specific surface and porosity analyzer (Kubo 1000) so as to control the powder particles with approximately the same average particle size. Furthermore, 3 g glass particles and 80 mL of deionized water were added to the reactor for the test [39]. The leaching liquid was collected at the same time of day on days 1, 3, 7, 14, and 28 for elemental analysis by an iCPA6500 (ICP-OES). The normalized leaching rate $LR_{i}$ (g/(m^2^·d)) can be calculated by the following equation:

(1) $$LR_{i}=\frac{C_{i}\cdot V}{f_{i}\cdot S\cdot \Delta t}$$

where $C_{i}$ is the concentration of the element *i* in the leachate; $f_{i}$ is the fraction of the element present in the glass samples; Δt is the test time; and S/V is the surface-area-to-volume ratio of glass powders, which was chosen to be 2000 m^−1^ based on the standard.

## 3. Results and Discussion

### 3.1. Glass Forming and Crystallization Analysis

**Figure 1:**
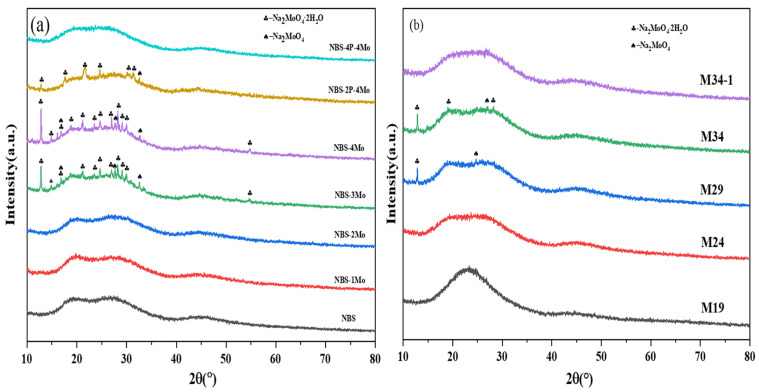
XRD patterns of (**a**) NBS-xMo and NBS-yP-4Mo series and (**b**) Mx series.

In the NBS-xMo series, with x representing the mol% MoO_3_, NBS and NBS-1Mo samples were colorless and transparent; NBS-2Mo and NBS-3Mo glass all presented a slight opalescent trail; and NBS-4Mo glass was opalescent and opaque. The NBS-yP-4Mo series presented an opalescent trail except for the NBS-4P-4Mo, with y representing the mol% P_2_O_5_. Figure 1a displays the XRD patterns of the NBS-xMo series and NBS-yP-4Mo series. For the NBS-xMo glass, NBS-1Mo and NBS-2Mo samples were XRD amorphous. With increasing MoO_3_ content, the XRD diffraction peaks of Na_2_MoO_4_·2H_2_O (PDF#34-0076) and Na_2_MoO_4_ (PDF#20-1130) in NBS-3Mo and NBS-4Mo could be observed. The presence of Na_2_MoO_4_·2H_2_O may have been due to the water affinity of anhydrous sodium molybdate, which may have adsorbed water from the air during the cooling process [40]. Notably, NBS-2Mo also presented a slight opalescent trail. The corresponding parent NBS glass was clear, so it is assumed that the opalescent trail was due to the presence of Na_2_MoO_4_. However, it lacked diffraction peaks corresponding to XRD, so their examination by XRD could not provide conclusive evidence when a crystalline phase presented in very minute quantities below the detection limit of XRD. For this reason, Raman spectra with higher detection sensitivity combined with XRD were chosen to analyze the S_Mo_ and crystallization. The XRD patterns of NBS-4Mo, NBS-2P-4Mo, and NBS-4P-4Mo showed gradually decreased phases of Na_2_MoO_4_ (PDF#20-1130). According to the variation of Na_2_MoO_4_ crystalline peaks in the NBS-yP-4Mo series, the effectiveness of P in inhibiting the growth of the Na_2_MoO_4_ crystalline phase could be inferred. NBS-4P-4Mo was the first sample to show an XRD amorphous state after phosphorus addition, so it was chosen as the reference glass of the Mz series, with the same composition as M24.

The Mz series were all translucent except for M29 and M34, which presented a slight opalescent trail. Zhou et al. studied Mo loading in conventional borosilicate glass and showed a higher depolymerized glass structure and more depolymerized regions in the glass structure with the substitutions of cationic oxides for SiO_2_, which could provide more sites to accommodate the [MoO_4_]^2−^ and the cations [17]. However, when Mo is present in the right proportion of the phosphate network through P–O–Mo bonds in phosphoborosilicate glass, it is another situation. Figure 1b displays the XRD patterns of the Mz series. M19 and M24 glass were found to be XRD amorphous. Gradually increased phases of Na_2_MoO_4_ (PDF#20-1130) and Na_2_MoO_4_·2H_2_O (PDF#34-0076) were observed in samples M29 and M34. When P was added to 8 mol based on M34, M34-1 was XRD amorphous.

### 3.2. Raman Spectra

**Figure 2:**
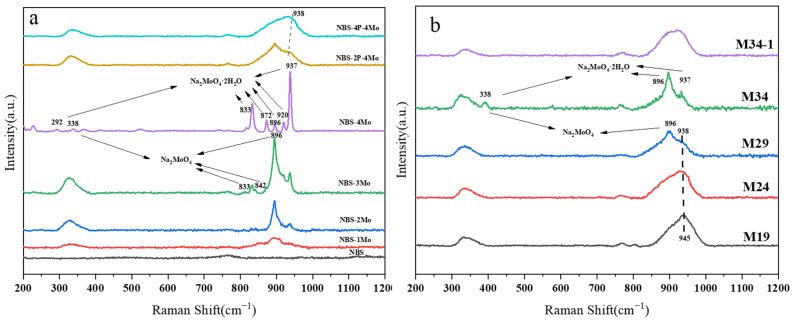
Raman spectra of (**a**) NBS-xMo and NBS-yP-4Mo series and (**b**) Mz series.

According to a review of the literature and reasonable analysis, the Raman band assignments for the samples used here are summarized in Table 2. The normalized Raman spectra of samples from the NBS-xMo series and NBS-yP-4Mo series are shown in Figure 2a. For the NBS-xMo glass, it was observed that increasing MoO_3_ induced systematic changes in Raman spectra. Specifically, the broad band of the NBS sample around 750–775 cm^−1^ was due to the six-membered borate rings with one or two BO_4_ [41]. The broad bands of NBS-1Mo were mainly located at ~900 and ~322 cm^−1^, indicating the symmetric stretching and bending vibrations of Mo–O of the [MoO_4_]^2−^ units within the glass structure, respectively [15,17]. The sharp bands at 338, 896, and 937 cm^−1^ of NBS-2Mo corresponded to characteristic features of the [MoO_4_]^2−^ in Na_2_MoO_4_, indicating the formation of Na_2_MoO_4_ and Na_2_MoO_4_·2H_2_O [42,43]. This is consistent with the results of visual inspection. With the increase of MoO_3_ content, the sharp bands at 337, 896, and 937 cm^−1^ of NBS-3Mo and NBS-4Mo became significantly stronger, indicating that crystalline Na_2_MoO_4_ increased. For the samples in the NBS-yP-4Mo series, characteristic features of Na_2_MoO_4_ in the Raman spectra were gradually suppressed with increasing phosphate content. The sharp band of 896 cm^−1^ in NBS-2P-4Mo and NBS-4P-4Mo gradually disappeared, and the sharp bands of 338 and 937 cm^−1^ changed to broad bands, indicating the formation of the P–O–Mo bonds replacing [MoO_4_]^2−^ in Na_2_MoO_4_ [44]. In other words, the successful integration of Mo into glass network occurred through P–O–Mo bonds instead of only forming isolated [MoO_4_]^2−^. For the NBS-4P-4Mo sample, the absence of sharp bands indicated the absence of crystallization.

The normalized Raman spectra of samples from the Mz series are shown in Figure 2b. Similar to the M24 sample (NBS-4P-4Mo), the M19 lacked any sharp bands characteristic of the crystalline phase, confirming the amorphous characteristics of the samples. The sharp bands of M29 and M34 at 338, 896, and 937 cm^−1^ were correlated with the Na_2_MoO_4_ and Na_2_MoO_4_·2H_2_O crystalline phase with gradually increasing intensity, indicating that the crystalline tendency of Na_2_MoO_4_ was enhanced with “z” values, which is consistent with the results of XRD data. Moreover, the broad band at ~938 cm^−1^ represented the gradual decrease of P–O–Mo with the Z value from 19 to 29.

### 3.3. ^31^P MAS NMR Spectroscopy

**Figure 3:**
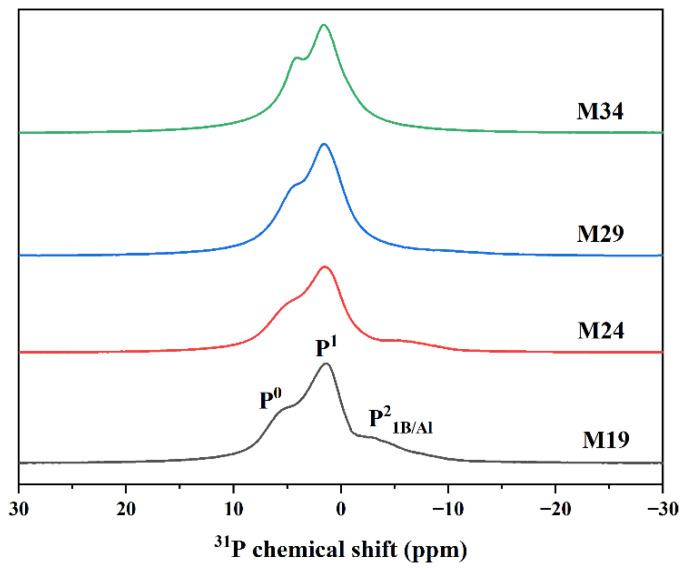
^31^P MAS NMR spectra of Mx series.

Figure 3 shows the ^31^P MAS NMR spectra of the Mz series, illustrating the spectral changes associated with increasing the Z value. Three main resonances were present in these spectra based on documented ^31^P chemical shifts, including a broad resonance centered near 1.5 ppm, a resonance appearing around 4–6 ppm, and a broad resonance between −7 and −4 ppm. The resonance near 5 ppm and 1.5 ppm were from isolated orthophosphate species (P^0^) and diphosphate species (P^1^) within the glass structure [18,31], respectively.

The mixed-network nature of glass implied that some “metaphosphate” units were likely to be bonded to other network formers, which may have modified the ^31^P chemical shifts. For example, A small amount of Al was found in the glass composition tests in Table 3, and some literature indicates that corundum crucible material was dissolved into the silicophosphate glass rather than any ion exchange occurring between the two materials [45,46]. In addition, the best agreement with the nominal composition was found for the sample melted in a platinum crucible. This phenomenon was not obvious due to the low phosphorus content in the Mx series and because the difference in Al content in the samples was not significant except for M34-1. The chemical shift between −7 and −4 ppm in Table 4 fell between a pyrophosphate (P–O–B/P–O–Al) anchored to a borate or an aluminate unit; however, it was not possible to identify contributions to ^31^P signals from P–O–B or P–O–Al bonds based on the chemical shift alone [18,27].

**Figure 4:**
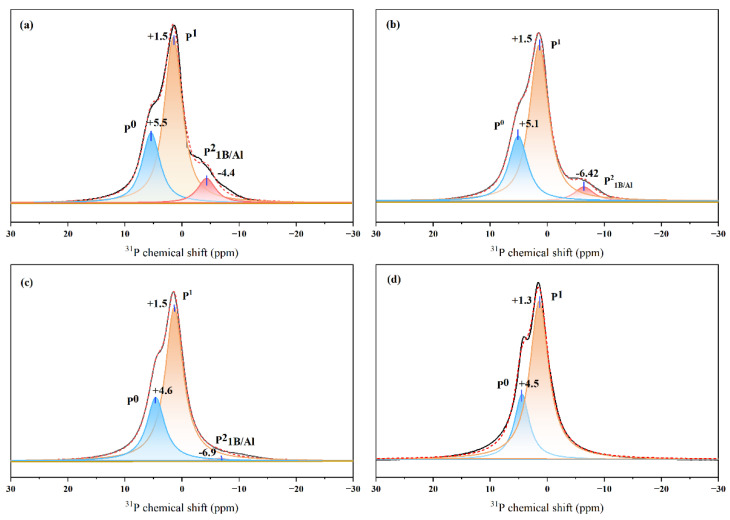
Deconvolution of the ^31^P MAS NMR spectra of (**a**) M19 (**b**) M24 (**c**) M29 (**d**) M34 samples.

Figure 4 shows that as the z value increased, i.e., SiO_2_ was progressively substituted by Na_2_O, an increase in the content of P^0^ and P^1^ occurred within the glass structure at the expense of P–O–B/P–O–Al bonds. Furthermore, the results are listed in Table 3. Interestingly the Raman spectra showed that P–O–Mo bonds also decreased with the value “z”. Arun Krishnamurthy et al. proposed a model for the network structure of phosphorus–molybdenum–borosilicate glass, where a portion of P–O–B bonds are probably bonded to P–O–Mo bands, which is consistent with the above phenomenon [27]. The crystalline molybdate phase (Na_2_MoO_4_) increased with the “z” value in the Mz series in this work. The results indicated that due to the variation of phosphate species, the effect of P on inhibiting the growth of the Na_2_MoO_4_ crystalline phase may decrease with increasing z values.

### 3.4. Thermal Stability and Aqueous Durability

**Figure 5:**
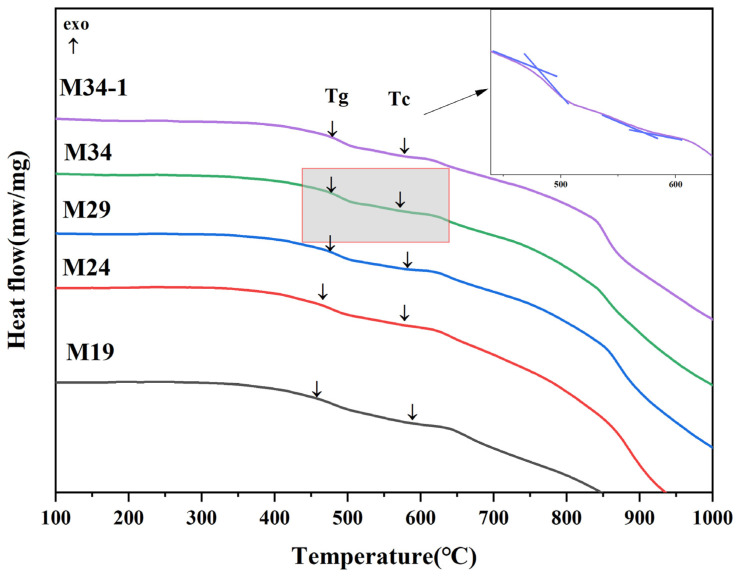
DSC curve of Mz series.

**Figure 6:**
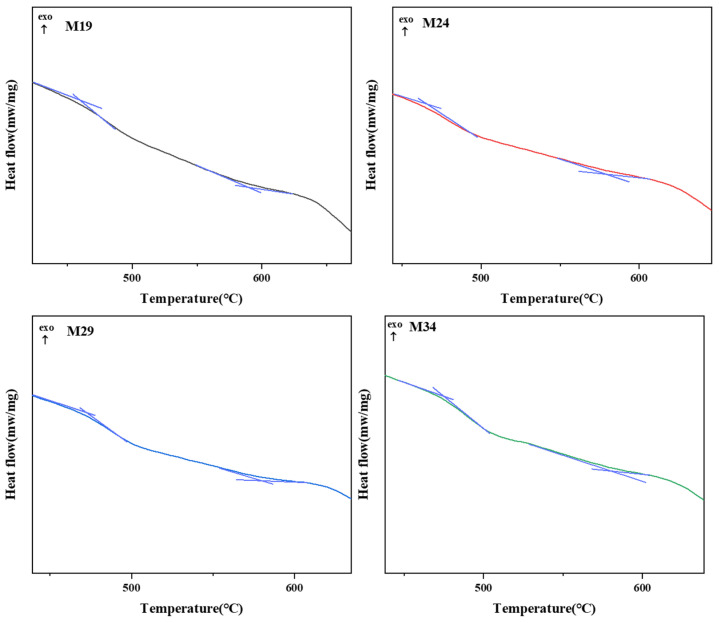
DSC curve zoom area of Mz series.

As thermal stability and chemical stability is highly significance for the safety assessment of nuclear glass, the aqueous durability and thermal stability of some samples in Mz series were tested and compared for further exploration. DSC curves of the selected samples are shown in Figure 5 and Figure 6. The glass transition temperature (*T_g_*) values of M19, M24, M29, M34, and M34-1 were 460 °C, 466 °C, 473 °C, 475 °C, and 475 °C, respectively. The glass crystallization onset temperature (*T_c_*) values of M19, M24, M29, M34, and M34-1 were 590 °C, 578 °C, 581 °C, 573 °C, and 579 °C, respectively. Lower (*T_c_*−*T_g_*) may lead to glass crystallization [47]. M34 had the lowest (*T_c_*−*T_g_*) values listed in Table 5, which may be related to the higher number of the Na_2_MoO_4_ crystalline phase of M34.

**Figure 7:**
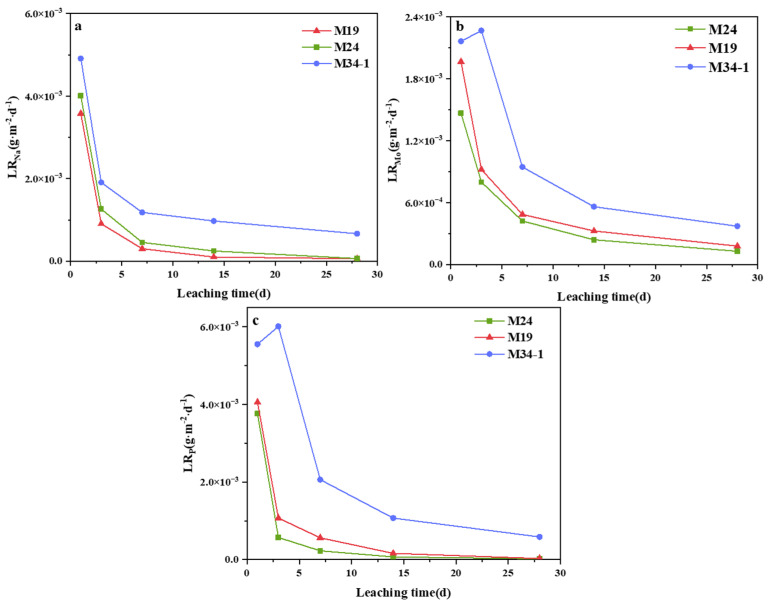
Normalized leaching rates of (**a**) Na, (**b**) Mo and (**c**) P.

Figure 7 shows the computed results of the normalized leaching rates of Na (*LR*_Na_), P (*LR*_P_), and Mo (*LR*_Mo_). Generally, the leaching rate of most samples dropped drastically with immersing time for the initial 7 days, and then the decreasing rates slowed down, and even basically remained unchanged after 14 days. The series of M19 and M24 were consistent with the above. However, a continuous increase in the *LR*_Mo_ of M34-1 was observed before 3 days, which may have been due to the effect of P on the glass structure.

This paper aims to study the effect of phosphorus content on the solubility of molybdenum in borosilicate glass and to help researchers to assess the doping proportion and effectiveness of P in borosilicate glass for improving Mo vitrification. We found that samples M19 and M24 showed similar trends, though they had different SiO_2_/B_2_O_3_ and Na_2_O/B_2_O_3_ ratios. Combined with the anomalous behavior of M34-1, this may indicate that low levels of phosphate doping do not unduly compromise the chemical durability of M19 and M24. Moreover, the highest leaching rate was observed for sample M34-1, indicating that excessive levels of P doping may compromise the matrix. The normalized leaching rates of all samples after 28 days listed in Table 5 met the nuclear industry standards (<1 g·m^−2^·d^−1^) [48].

## 4. Conclusions

In the present work, the addition of phosphate to NBS glass increased the solubility of Mo, and NBPS glass could accommodate up to 4 mol% MoO_3_ without the presence of crystalline molybdates having been successfully synthesized. According to Raman spectra and NMR results, the effectiveness of phosphate may be related to the Na content in borosilicate glass. It is anticipated that a high Na content borosilicate glass matrix requires more P doping to accommodate Mo. Interestingly, Zhou et al. proposed that the substitutions of cationic oxides for SiO_2_ improve S_Mo_ in borosilicate glass [17]. However, the results may not be consistent in the presence of P elements in a borosilicate glass matrix. In addition, incorporating large amounts of P can compromise the aqueous durability of the glass matrix.

## Figures and Tables

**Table 1:** Composition of glasses in the present study (mol%).

Samples	SiO_2_	B_2_O_3_	Na_2_O	P_2_O_5_	MoO_3_
NBS	53.00	21.00	26.00		
NBS-1Mo	52.47	20.79	25.74		1
NBS-2Mo	51.94	20.58	25.48		2
NBS-3Mo	51.41	20.37	25.22		3
NBS-4Mo	50.88	20.16	24.96		4
NBS-2P-4Mo	49.82	19.74	24.44	2	4
NBS-4P-4Mo	48.76	19.32	23.92	4	4
M19	53.76	19.32	18.92	4	4
M24	48.76	19.32	23.92	4	4
M29	43.76	19.32	28.92	4	4
M34	38.76	19.32	33.92	4	4
M34-1	37.07	18.48	32.45	8	4

**Table 2:** Assignments of the bands in the observed Raman spectra.

	Assignment	References
Broad 750–775	Six-membered borate rings containing BO_4_ tetrahedra	[41]
Broad ~322	Bending vibration of [MoO_4_]^2−^	[17]
Broad ~900	Symmetric stretching vibration of [MoO_4_]^2−^	[15]
Broad 938–954	vibration modes of Mo-O terminal bonds of Mo-O-P	[44]
Sharp 338, 833, 842, 896	Vibration modes of the [MoO_4_]^2−^ of Na_2_MoO_4_	[42]
Sharp 292, 833, 872, 896, 920, 937	Vibration modes of the [MoO_4_]^2−^ of Na_2_MoO_4_ 2H_2_O	[43]

**Table 3:** Nominal (measured) compositions (mol%) of the glass samples (M19, M24, M29, M34, and M34-1 samples).

Samples	SiO_2_	Na_2_O	Al_2_O_3_	P_2_O_5_	MoO_3_
M19	66.63(64.92)	23.45(21.79)	0(2.72)	4.96(4.64)	4.96(5.91)
M24	60.44(58.83)	29.65(27.77)	0(2.68)	4.96(4.62)	4.96(6.09)
M29	54.24(51.86)	35.85(34.54)	0(2.76)	4.96(4.60)	4.96(6.21)
M34	48.04(45.94)	42.04(40.36)	0(2.70)	4.96(4.67)	4.96(6.24)
M34-1	45.47(43.32)	39.84(36.15)	0(5.01)	9.81(9.23)	4.91(6.27)

Note: B_2_O_3_ is not included in the nominal compositions in this table because B cannot be detected by XRF.

**Table 4:** ^31^P MAS NMR fitting parameters (Chemical shift and relative area A).

Sample	Chemical Shift (ppm)	Phosphate Units	A (%)	Ref.
M19	+5.5	P^0^	27.40	[18,27,31,33]
	+1.5	P^1^	61.35	
	−4.4	P^2^_1B/Al_	11.25	
M24	+5.1	P^0^	28.38	
	+1.5	P^1^	61.99	
	−6.4	P^2^_1B/Al_	9.63	
M29	+4.6	P^0^	29.16	
	+1.5	P^1^	67.62	
	−6.9	P^2^_1B/Al_	3.22	
M34	+4.5	P^0^	24.95	
	+1.3	P^1^	75.05	

**Table 5:** Thermal parameters and leaching rates of the samples in the Mz series.

Samples	M19	M24	M29	M34	M34-1
*T_g_* (°C)	460	466	473	475	475
*T_c_* (°C)	590	578	581	573	579
*T_c_*−*T_g_* (°C)	130	112	108	98	104
Na (*LR*_Na_)/ × 10^−4^ g·m^−2^·d^−1^ (28 d)	6.04	6.59	/	/	6.65
Mo (*LR*_Mo_)/ × 10^−4^ g·m^−2^·d^−1^ (28 d)	1.80	1.29	/	/	3.73
P (*LR*_P_)/ × 10^−4^ g·m^−2^·d^−1^ (28 d)	2.21	3.08	/	/	5.83

## Data Availability

Not applicable.
